# A Case of a Tarlov Cyst in a Pediatric Patient With Ehlers-Danlos Syndrome

**DOI:** 10.7759/cureus.29009

**Published:** 2022-09-10

**Authors:** Shadi Shams, Riddhima Issar, Tanya Kadrmas-Iannuzzi

**Affiliations:** 1 Medicine, Rowan School of Osteopathic Medicine, Stratford, USA; 2 Internal Medicine, Jefferson Health Washington Township, Turnersville, USA; 3 Internal Medicine, Rowan School of Osteopathic Medicine, Stratford, USA; 4 Pediatric Medicine, Rowan School of Osteopathic Medicine, Stratford, USA

**Keywords:** urinary incontinence, tarlov cyst, recurrence, postoperative complications, ehlers-danlos syndrome

## Abstract

Ehlers-Danlos Syndrome (EDS), a rare genetic disorder, causes hyperlaxity, skin bruising, vascular disruption, and organ rupture. It presents with numerous complications, ranging from delayed gastric emptying to spontaneous rupture of blood vessels. A rare complication involves the neurological system and causes Tarlov cysts in the spinal canal. This gives rise to several symptoms, ranging from urinary and bowel incontinence to numbness and paresthesia. We report a case of an 11-year-old male with a past medical history of Ehlers-Danlos Syndrome, who presented with continued urinary and bowel incontinence, which was eventually found to be due to a Tarlov cyst. Although a handful of reports of Tarlov cysts exist in the literature, a presentation in a pediatric patient with a history of Ehlers-Danlos Syndrome is unconventional and unforeseen.

## Introduction

Ehlers-Danlos Syndrome (EDS) is a rare genetic disorder of the body’s connective tissue [1]. This disease occurs due to mutations in the synthesis and processing of collagen, which has various manifestations such as the fragility of the skin, ligaments, joints, and blood vessels [1]. Clinical symptoms can range from hyperlaxity and physical disability to life-threatening vascular disease [1]. The incidence of EDS is estimated to be between 1 in 2500 and 1 in 5000 in the general population and 1 in 5000 in the pediatric population [2,3]. The most common subtype is hypermobility, with an incidence of 1 in 10,000 to 1 in 15,000 [3]. It is hard to make an accurate estimation, as many individuals with milder presentations rarely seek medical help [3]. Due to joint mobility, patients with EDS are more prone to trauma during everyday activities. Regardless of the subtype, morbidity in EDS depends mostly on the patient’s environmental factors [3]. Some subtypes, such as hypermobile and classic, have a mild disease course that does not influence their mortality rate [3]. On the other hand, in vascular and kyphoscoliotic subtypes, the lifespans are significantly affected by vascular insults and lung restriction [3]. Neurological complications like musculoskeletal pain, fatigue, muscle weakness, dystonia, and paresthesia have been noted [4]. Other severe complications are idiopathic intracranial hypertension, migraine, tethered cord syndrome, and Chiari malformation type 1 [5].

Another rare neurological complication is the spinal Tarlov cyst, which is caused by the weakening of the epineurium and perineurium in EDS [5]. Tarlov cysts are incidental radiological findings that appear at the level of the sacrum [5]. These cysts are often asymptomatic but can produce symptoms of pain and neurological deficits due to nerve root compression [5]. Some common symptoms are sacral pain, sciatica, and bowel and bladder dysfunction [5]. Tarlov cysts in the pediatric population are a rare phenomenon. A large meta-analysis demonstrated a 0.53% incidence of Tarlov cysts in the pediatric population [6]. The study also delineated that Tarlov cysts are more prominent in the female sex [6]. A few cases have shown an association between Tarlov cysts and EDS. One of which is the case of a 40-year-old female with EDS, who had multiple meningeal cysts in her sacral spinal cord [7]. However, little to no cases have reported the incidence of spinal cysts in the pediatric population with EDS. Some treatments for Tarlov cysts are surgical removal, fibrin glue injection, and cyst aspiration [5]. Research has shown that Tarlov cyst surgical obliteration is 80-88% successful, and patients have complete resolution of symptoms postoperatively [5]. This case presents the rare phenomenon of a Tarlov cyst in a pediatric patient with EDS. It further delves into the postoperative recurrence of symptoms.

## Case presentation

**Figure 1:**
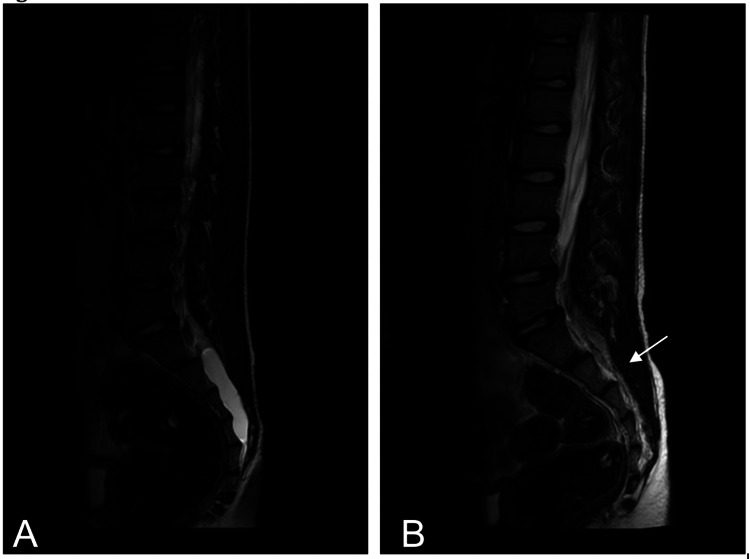
MRI T2 lumbar spine - sagittal view A represents the preoperative scan (five months prior to surgery) and B is the postoperative scan (four months after surgery) that reveals the complete resolution of the cyst and lack of recurrence. The arrow indicates the location of the tethered cord.

An 11-year-old male with a past medical history of Ehlers-Danlos Syndrome (hypermobile-type III) and CPA1 gene mutation initially presented at age nine with urinary incontinence and nocturnal enuresis. Successful toilet training was achieved at age four; however, the patient started having occasional (twice-a-month) fecal accidents a year later. From age seven to nine, the patient had frequent urinary and fecal incontinence, which were initially treated pharmacologically with oxybutynin. Upon investigation, he was found to have a tethered spinal cord and a Tarlov cyst in the distal spinal cord. The Tarlov cyst initially was not picked up by imaging, and it required narrowing the fields for the lesion to be detected. As shown in Figure 1A, preoperative MRI of the lumbar spine without contrast showed a cystic lesion filling the distal spinal canal at the level of S1/S2 with mild thickening of the distal filum terminale. The characteristics of the cystic lesion were diagnostic of a perineural (Tarlov) cyst. Due to the worsening severity of symptoms, the patient underwent cyst removal and S1 and S2 laminectomy in May 2020 at age 10. Post resection, the patient's urinary and bowel incontinence symptoms improved drastically and continued to improve at the six-month mark. As shown in Figure 1B, lumbar MRI without contrast four months post-surgery showed no new intraspinal lesions and no new meningeal cysts.

Currently, at age 11, the patient has had a recurrence of urinary incontinence up to five times a week and exacerbated by daily activities. Additionally, the patient reported lower back pain and numbness. A repeat MRI of the lumbar spine from August 2021 showed no recurrence of the treated cyst. However, his surgeon noticed a thin line on the MRI that was not visualized earlier, indicating the presence of a tethered cord in the same region. The patient underwent another lumbar laminectomy for microsurgical resection of the filum terminale in the tethered cord. Postoperatively, the patient endorsed improvement with mobilities, reduction in pain, paresthesia in lower extremities, and radiating pain down his legs. He continues to have bowel and bladder incontinence and intermittent nerve pain, managed with Tamsulosin 0.4 mg, Pregabalin 100 mg, and Hydromorphone as needed.

## Discussion

This case delineates the significance of recognizing the diagnosis of the Tarlov cyst in children with a history of EDS. This is especially important, as the current literature has little evidence of the prevalence of Tarlov cysts in conjunction with EDS [8].

Many cases of adult patients have shown Tarlov cysts to be a complication of EDS [7,9-12], and only one such shows this association in a pediatric patient [11]. Our case adds to this limited data and helps recognize urinary incontinence as a symptom of Tarlov cyst development. As previously mentioned, one of the main treatment modalities for the Tarlov cyst is surgical removal in the pediatric population [9,12]. This is an effective method for reducing radiculopathies and bowel and bladder symptoms with low recurrence [9,12]. Although, in this case, the patient's condition improved up to a year postoperatively, the symptoms recurred shortly after. Despite the surgical intervention, the patient continues to have bowel and bladder dysfunction. This raises the question of whether earlier diagnosis and treatment could have prevented the progression of symptoms and deterioration.

## Conclusions

This case presentation varies vastly from most cases reported in the literature due to the atypical occurrence of the Tarlov cyst in a pediatric patient with a history of EDS. Therefore, it is imperative to keep the Tarlov cyst as a differential in children with EDS who present with chronic urinary and bowel incontinence. We recommend screening patients with EDS for symptoms of incontinence, lower extremity paresthesia, and pain to recognize underlying spinal cord involvement. Identifying the disease early on can help intervene medically or surgically to ameliorate symptom severity. This paper attempts to expand the current literature on the diagnosis of EDS and its rare complication of Tarlov cysts in the pediatric population.
